# Phenotype-genotype correlation in multiple primary lung cancer patients in China

**DOI:** 10.1038/srep36177

**Published:** 2016-10-31

**Authors:** Yang Yang, Wei Yin, Wenxin He, Chao Jiang, Xiao Zhou, Xiao Song, Junjie Zhu, Ke Fei, Weijun Cao, Gening Jiang

**Affiliations:** 1 Department of Thoracic Surgery, Tongji University Shanghai Pulmonary Hospital, Shanghai 200433, China; 2 Key Laboratory of Oral Biomedical Engineering of Ministry of Education, Hospital and School of Stomatology, Wuhan University, Wuhan 430079, China; 3 Department of Respiratory, Tongji University Shanghai Pulmonary Hospital, Shanghai 200433, China

## Abstract

Due to recent advances in high-resolution detection technology, multiple primary lung cancer (MPLC) is becoming an increasingly common diagnosis. However, the genotype-phenotype correlations in MPLC patients have not yet been assessed. In this study, we analyzed the clinical and pathological data for 129 consecutive MPLC patients who received curative surgery at the Tongji University Shanghai Pulmonary Hospital, China. We have screened 129 patients in the present study and found mutations in *EGFR, BRAF, ROS1* and *KRAS* genes, as well as the rearrangement of the *EML4-ALK* gene in 113 patients. The mean patient age was 59.9 (25–78) years old and 41 patients were males (31.8%). Among the total patients, 123 (95.4%) had two primary lesions, 5 (3.9%) had three primary lesions, and 1 (0.8%) had four primary lesions. In 38.8% of the patients, all lesions were located on only one side of the body. Most of the detected mutations (98 patients) were in the *EGFR* gene. The patients exhibited significant differences in the *EGFR* mutation, age at diagnosis, and foci location.

Multiple primary lung cancer (MPLC) refers to the synchronous or metachronous appearance of more than one primary lung cancer in a single patient. MPLC was first reported in 1924^1^. In recent years, the rate of diagnosis worldwide has rapidly increased due to the improvement in radiological diagnostic procedures such as high-resolution multi-slice spiral computed tomography (CT) and positron emission tomography (PET)^2^,^3^,^4^,^5^. However, preoperative diagnosis can be problematic because it is often difficult to differentiate MPLC from lung cancer metastasis based on radiological and histopathological criteria alone.

Identification of oncologic biomarkers by molecular genetic analysis may aid in the accurate diagnosis of MPLC. Potential oncologic biomarkers for analysis include allelic loss of heterozygosity (LOH) markers and specific gene mutations^5^,^6^,^7^,^8^,^9^,^10^,^11^,^12^. Recent advances in tumor molecular biology have resulted in the identification of several candidate markers such as *P53*^13^ and the *EGFR* gene^14^ that can be used for MPLC diagnosis. In addition, several genes have been confirmed to be driver genes in non-small cell lung cancer (NSCLC), including anaplastic lymphoma receptor tyrosine kinase (*ALK*), v-akt murine thymoma viral oncogene homolog 1 (*AKT1*), B-Raf proto-oncogene, serine/threonine kinase (*BRAF*), v-erb-b2 avian erythroblastic leukemia viral oncogene homolog 2 (*ERBB2*), Kirsten rat sarcoma viral oncogene homolog (*KRAS*) and phosphatidylinositol-4,5-bisphosphate 3-kinase, catalytic subunit alpha (*PIK3CA*)^15^,^16^,^17^,^18^,^19^,^20^. However, current knowledge of the molecular characteristics of MPLC is insufficient to allow for validation of the accuracy and sensitivity of molecular diagnosis. In this study, we screened for mutations in several oncogenic driver genes in a cohort of Chinese MPLC patients and analyzed the data to correlate our genetic findings with the clinical phenotypes.

## Materials and Methods

### Ethical approval

This study was conducted in accordance with the amended Declaration of Helsinki, and it was approved by the Institutional Review Board (IRB) of Tongji University Shanghai Pulmonary Hospital, China. Written informed consent was obtained from all participants in this study. The methods were carried out in accordance with the approved guidelines.

### Patients and specimen collection

From May 2011 to January 2015, 129 consecutive NSCLC patients with MLPC received surgical resection of their lung lesions at the Shanghai Pulmonary Hospital. Post-operative histological analyses confirmed the presence of primary lung cancer in all patients. Fresh primary tumor tissues containing more than 50% tumor cells were collected from all patients during surgery and were used for subsequent gene mutation analyses.

Demographic and clinical data were collected from the patient notes and computer records, including age, gender, the tumor size and location, the TNM stage and the histological type.

### Candidate gene mutation analysis

Genomic DNA and the total RNA were immediately extracted from fresh tissues using a commercial QIAamp DNA Tissue Kit and RNeasy Kit (Qiagen, Germany), respectively. Mutations in the *EGFR, BRAF, ROS1* and *KRAS* genes as well as the rearrangement of *EML4-ALK* were detected with Amoy Diagnostics kits (Xiamen, China) utilizing proprietary real-time PCR technology to detect mutations in the target genes.

### Statistical analysis

Statistical analysis was performed using SPSS software (Version 18.0, Chicago, IL). The Chi-square tests and one-way ANOVA were used to detect associations between the clinical characteristics and studied genes. A P value of <0.05 was regarded as being significant.

## Results

### Demographic and Clinical characteristics

**Figure 1:**
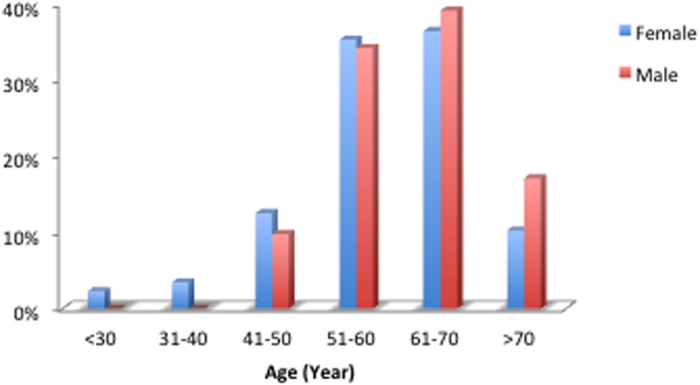
The age distribution of the 129 MPLC patients analyzed.

**Figure 2:**
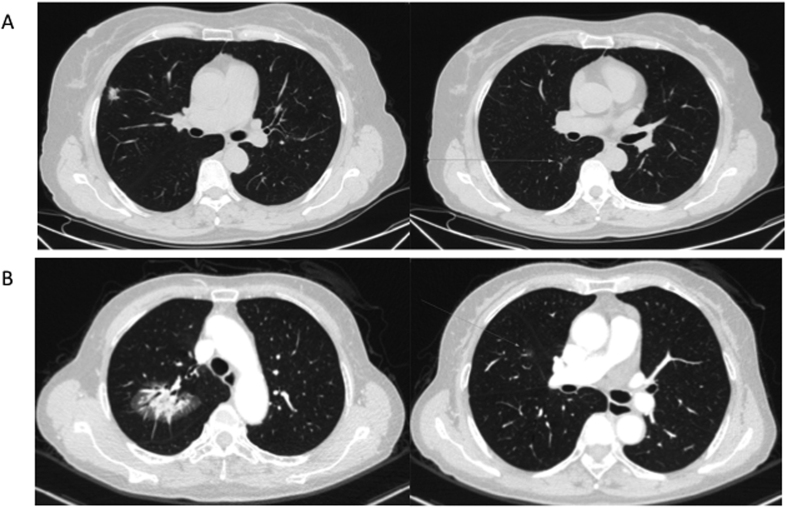
CT scan images for two MPLC patients. (**A**) Images of Patient I. Left, CT scan of upper right lobi pulmonis; and right, CT scan of lower right lobi pulmonis. (**B**) Images of Patient II. Left, CT scan of upper right lobi pulmonis; and right, CT scan of middle right lobi pulmonis.

**Figure 3:**
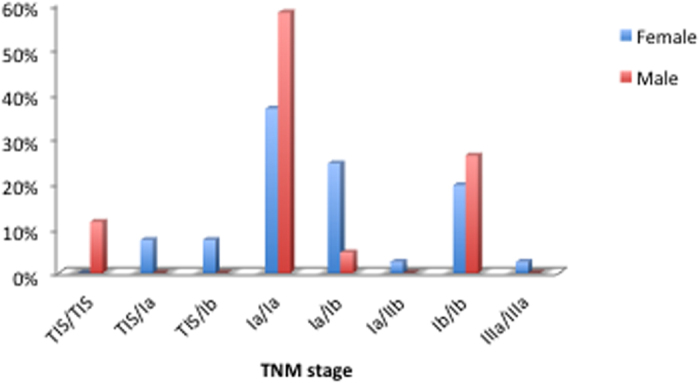
The TNM stages profile of these MPLC patients.

This cohort study included 41 male and 88 female patients,. The mean age was 59.9 (25–83) years old (Fig. 1). All the 5 youngest patients (<40 years) were females. A total of 299 foci were removed during the surgery. All lesions were detected simultaneously in each patient (Fig. 2). In total, 123 patients (95.35%) had two lesions, 5 patients (3.88%) had three lesions and 1 patient (0.78%) had four lesions. In 50 patients (38.8%), all the tumors were located on the same side. Most of the tumors were detected at an early stage, including 26 foci (8.70%) at the Tis stage (Fig. 3). Multiple lesions with an identical histological type were recorded in 88 patients, whereas multiple lesions with different histological types were detected in the other 41 patients.

### Mutation spectrum

Mutations in the *EGFR, KRAS, BRAF* and *ROS1* genes and the rearrangement of *EML4-ALK* were detected in 113 patients (87.6%). Most of the patients (98) had *EGFR* mutations. No mutations were detected in 8 (19.5%) male and 8 (9.1%) female patients, and mutations in two genes were identified in 10 patients (Tables 1, 2, 3 and 4). A total of 12 subtypes of *EGFR* gene mutations were identified, including 6 types of complicated mutations. Complicated mutations (L858R/S768I, L858R/20ins, G719X/T790M, 19del/T790M, 19del/L858R and 19del/20ins) were only found in female patients, whereas the mutation spectrum of the male patients was relatively simple. Only exon 19del and L858R mutations were detected in the male patients (Tables 5 and 6).

### Phenotype-genotype correlation in MPLC patients

After determining the MPLC patient genotypes, we analyzed the phenotype-genotype correlations among these individuals. Significant differences were detected between the presence of *EGFR* mutations and the age at diagnosis and foci location (Table 7). Rare *EGFR* mutations (G719X, S768I, T790M and L861Q) were detected in 18 female patients. These rare mutations were accompanied by common mutations (L858R, exon 19del and exon 20ins) in multiple lesions. Among the lesions with L858R and a rare mutation, 6.25% were mucinous adenocarcinomas, whereas 62.5% were invasive adenocarcinomas. Further, 31.25% of these lesions were located in left upper lobe. Notably, none of the young patients had these rare mutations.

## Discussion

The etiology of MPLC remains unclear. At present, the field cancerization theory is the most common explanation^12^. The accurate preoperative diagnosis of MPLC can be problematic. Hitherto, differentiating between MPLC and lung metastases in patient with multi-focal lung cancer has mainly depended on morphological methods. The diagnostic criteria for MPLC recommended by the American College of Chest Physicians (ACCP) are as follows: (1) the presence of anatomically separated tumors with the same histology, with the cancers in different lobes, no N2 or N3 involvement, and no systemic metastases; (2) the presence of temporally separated tumors with the same histology, with a 4-yr interval between cancers and no systemic metastases from any of the cancers; and (3) the presence of tumors with different histologies, including tumors with different histologic types and different molecular genetic characteristics or tumors arising separately from *in situ* carcinoma foci^21^,^22^,^23^.

In spite of these difficulties, it remains critical to distinguish between primary and metastatic lung cancer because the long-term survival of these two types of cancer is significantly different. Metastatic lung cancers, especially in patients with multiple metastases, should not be resected as the prognosis is extremely poor. On the other hand, most MPLC patients have separate lesions that are each individually at an early stage. The 5-year survival rate for synchronous MPLC after curative surgery could be as high as 75.8%^24^. Therefore, the proper diagnsis of MPLC is key to the appropriate treatment of patients.

Molecular genetic diagnosis was introduced into the diagnostic criteria for ACCP in 2013; however, the lack of effective molecular markers has limited its clinical application in practice. Hence, there is a pressing need for comprehensive phenotypic and genotypic analyses of MPLC patients.

Multiple factors can contribute to the development of MPLC, including genetic factors, smoking and exposure to environmental pollutants. Previous studies^3^ have shown that MPLC is more common in 50- to 70-year-old male smokers; most tumors in these patients have been reported to be squamous carcinoma-squamous carcinoma, squamous carcinoma-adenocarcinoma or adenocarcinoma-adenocarcinoma. In contrast with these data, our findings suggests that MPLC is more common in females, although they confirm that 50- to 70-year-old patients represent the highest-risk age group.

In our study, we screened for mutations in the *EGFR, BRAF, ROS1* and *KRAS* genes, as well as the rearrangement of the *EML4-ALK* gene.

Since the direct sequencing of nucleotides is a time-consuming and labor-intensive process^25^,^26^, we used a real-time PCR-based method to analyze these mutations. Both previous studies and our experience suggested that a real-time PCR-based method would detect the mutations of interest with the same efficiency as direct sequencing^27^,^28^,^29^,^30^. Only 13.95% of the patients screened were found to be free from any mutations in the genes analyzed in the present study. The frequency of *EGFR* gene mutations (76.1% in the female patients and 61.0% in the male patients) was higher in our study than that reported in Chinese lung cancer patients with a single focus^31^,^32^. Furthermore, only 30 (32.6%) of the MPLC patients had identical gene mutations. These data suggest that more than half of second primary lung cancers result from different mechanisms compared with primary cancers. In addition, a previous study indicated that the risk of second primary lung cancers in patients treated with surgical resection for stage I NSCLC is 1.99 per 100 patient-years^33^. Based on these results, we conclude that each MPLC lesion should be independently diagnosed and treated by surgical resection.

Our results suggested that rare mutations in the *EGFR* gene contributed significantly to the diversity of mutations detected in female patients. Although these data provide evidence of genotype-phenotype correlations in MPLC patients, further study involving more patients will be needed to confirm these conclusions.

## Additional Information

**How to cite this article**: Yang, Y. *et al*. Phenotype-genotype correlation in multiple primary lung cancer patients in China. *Sci. Rep.* **6**, 36177; doi: 10.1038/srep36177 (2016).

**Publisher’s note:** Springer Nature remains neutral with regard to jurisdictional claims in published maps and institutional affiliations.

## Figures and Tables

**Table 1:** The distribution of gene mutations among 129 patients.

	Male		Female	
*EGFR*	24	58.54%	64	72.73%
*KRAS*	7	17.07%	3	3.41%
*BRAF*	0	0.00%	3	3.41%
*ROS1*	0	0.00%	1	1.14%
*EML4-ALK*	1	2.44%	0	0.00%
*EGFR* + *KRAS*	1	2.44%	2	2.27%
*EGFR* + *EML4-ALK*	0	0.00%	6	6.82%
*EGFR* + *ROS1*	0	0.00%	1	1.14%
No mutation in the above genes	8	19.51%	8	9.09%

**Table 2:** Details of somatic mutations in *EGFR, BRAF* and *KRAS* genes analyzed in this study.

Gene	Mutation	Exon	Base Change
*EGFR*			
	G719A	18	2156G > C
	G719S	18	2155G > A
	G719C	18	2155G > T
	E746_A750del (1)	19	2235_2249del15
	E746_A750del (2)	19	2236_2250del15
	L747_P753 > S	19	2240_2257del18
	E746_T751 > I	19	2235_2252 > AAT(complex)
	E746_T751del	19	2236_2253del18
	E746_T751 > A	19	2237_2251del15
	E746_S752 > A	19	2237_2254del18
	E746_S752 > V	19	2237_2250 > T(complex)
	E746_S752 > D	19	2238_2255del18
	L747_A750 > P	19	2238_2248 > GC(complex)
	L747_T751 > Q	19	2238_2252 > GCA(complex)
	L747_E749del	19	2239_2247del9
	L747_T751del	19	2239_2253del15
	L747_S752del	19	2239_2256del18
	L747_A750 > P	19	2239_2248TTAAGAGAAG > C (complex)
	L747_P753 > Q	19	2239_2258 > CA (complex)
	L747_T751 > S	19	2240_2251del12
	L747_T751del	19	2240_2254del15
	L747_T751 > P	19	2239_2251 > C (complex)
	T790M	20	2369C > T
	S768I	20	2303G > T
	H773_V774insH	20	2319_2320insCAC
	D770_N771insG	20	2310_2311insGGT
	V769_D770insASV	20	2307_2308insgccagcgtg
	L858R	21	2573T > G
	L861Q	21	2582T > A
*KRAS*			
	Gly12Asp		GGT > GA T
	Gly12Ala		GGT > GCT
	Gly12Val		GGT > GTT
	Gly12Ser		GGT > AGT
	Gly12Arg		GGT > CGT
	Gly12Cys		GGT > TGT
	Gly13Asp		GGC > GAC
*BRAF*			
	V600E	15	1799T > A

**Table 3:** *EML4-ALK* fusions detected in this study.

Alternate name	*EML4* spliced exon	Breakpoint variety (bp)*	*ALK* spliced exon
*EML4-ALK* variant 1	13	−/−/ins69/−/−/−	20
*EML4-ALK* variant 3a/b	6	−/−/ins33/−	20
*EML4-ALK* variant 2	20	−/−/ins18/−	20
*EML4-ALK* variant 4	15	del71	20
*EML4-ALK* variant 4′	14	ins11del49/del12/del36	20
*EML4-ALK* variant 5′	18	—	20
*EML4-ALK* variant 5a/b	2	−/ins117	20
/	17	ins68	20

^*^Differing breakpoints result in different cDNA isoforms: ins indicates insertion, and del indicates deletion.

**Table 4:** *ROS1* gene fusions detected in this study.

Alternate name	Fused gene	*ROS1* spliced sites
*ROS1*-1	SLC34A2 exon 4	ROS1 exon 32
	SLC34A2 exon 4	ROS1 exon 34
*ROS1*-2	SLC34A2 exon 13	ROS1 exon 32
	SLC34A2 exon 13	ROS1 exon 34
*ROS1*-3	CD74 exon 6	ROS1 exon 32
	CD74 exon 6	ROS1 exon 34
*ROS1*-4	SDC exon 2	ROS1 exon 32
	SDC exon 2	ROS1 exon 34
*ROS1*-5	SDC exon 4	ROS1 exon 32
	SDC exon 4	ROS1 exon 34
	EZR exon 10	ROS1 exon 34
*ROS1*-6	TPM3 exon 8	ROS1 exon 35
	LRIG3 exon 16	ROS1 exon 35
*ROS1*7	FIG exon 8	ROS1 exon 35
	FIG exon 4	ROS1 exon 36

**Table 5:** The clinicopathological characteristics of the male MPLC patients.

	19del + L858R	19del + WT	L858R	L858R + WT	WT
Age					
<30	0.00%	0.00%	0.00%	0.00%	0.00%
<40	0.00%	0.00%	0.00%	0.00%	0.00%
<50	20.00%	33.33%	0.00%	0.00%	12.50%
<60	20.00%	33.33%	40.00%	57.14%	25.00%
<70	40.00%	33.33%	40.00%	42.86%	37.50%
<80	0.00%	0.00%	10.00%	0.00%	25.00%
>80	20.00%	0.00%	10.00%	0.00%	0.00%
Site					
Left	60.00%	33.33%	35.00%	42.86%	50.00%
Right	40.00%	66.67%	65.00%	57.14%	50.00%
Pathology					
Lung adenocarcinoma	90.00%	66.67%	95.00%	85.71%	81.25%
Squamous carcinoma	10.00%	0.00%	0.00%	0.00%	15.63%
Large cell carcinoma	0.00%	0.00%	0.00%	7.14%	3.13%
Atypical hyperplasia of adenoma	0.00%	33.33%	5.00%	7.14%	0.00%
TNM stage					
Tis	0.00%	0.00%	5.00%	14.29%	9.38%
Ia	70.00%	16.67%	60.00%	50.00%	53.13%
Ib	30.00%	83.33%	25.00%	35.71%	34.38%
IIb	0.00%	0.00%	0.00%	0.00%	3.13%
IIIa	0.00%	0.00%	10.00%	0.00%	0.00%

**Table 6:** The clinicopathological characteristics of the female MPLC patients.

	19del	19del + L858R	19del + rare mutation	19del + WT	L858R + rare mutation	L858R	L858R + WT	G719X	20ins + WT	WT
Age										
<30	0.00%	0.00%	0.00%	0.00%	0.00%	0.00%	8.33%	0.00%	0.00%	0.00%
<40	0.00%	0.00%	33.33%	0.00%	0.00%	0.00%	0.00%	0.00%	0.00%	10.53%
<50	0.00%	0.00%	33.33%	9.09%	0.00%	0.00%	16.67%	0.00%	0.00%	31.58%
<60	50.00%	25.00%	0.00%	36.36%	50.00%	23.08%	25.00%	100.00%	100.00%	42.11%
<70	50.00%	50.00%	0.00%	45.45%	33.33%	53.85%	41.67%	0.00%	0.00%	15.79%
<80	0.00%	25.00%	33.33%	9.09%	16.67%	23.08%	8.33%	0.00%	0.00%	0.00%
Site										
Left	33.33%	15.79%	50.00%	43.48%	43.75%	38.46%	42.31%	0.00%	0.00%	48.72%
Right	66.67%	84.21%	50.00%	56.52%	56.25%	61.54%	57.69%	100.00%	100.00%	51.28%
Pathology										
Adenocarcinoma	100.00%	100.00%	83.33%	78.26%	100.00%	100.00%	100.00%	100.00%	100.00%	89.74%
Squamous carcinoma	0.00%	0.00%	16.67%	0.00%	0.00%	0.00%	0.00%	0.00%	0.00%	0.00%
Atypical hyperplasia of adenoma	0.00%	0.00%	0.00%	21.74%	0.00%	0.00%	0.00%	0.00%	0.00%	10.26%
TNM stage										
Tis	0.00%	0.00%	0.00%	9.09%	0.00%	7.69%	0.00%	0.00%	0.00%	15.79%
Ia	66.67%	75.00%	66.67%	54.55%	83.33%	23.08%	91.67%	100.00%	100.00%	68.42%
Ib	33.33%	25.00%	33.33%	27.27%	0.00%	46.15%	8.33%	0.00%	0.00%	15.79%
Ia + Ib	0.00%	0.00%	0.00%	9.09%	16.67%	23.08%	0.00%	0.00%	0.00%	0.00%

**Table 7:** Correlation between *EGFR* mutations and clinicopathological features.

	*EGFR* mutation		*P* value
	Positive	Negative	
Gender			0.2035
Male	25	16	
Female	69	19	
Age			0.0236
<60	46	22	
≥60	48	13	
Location			0.0456
Left	73	35	
Right	109	36	
Pathology			0.2781
Adenocarcinoma	170	66	
Squamous carcinoma	2	0	
Large cell carcinoma	9	4	
Atypical hyperplasia of adenoma	1	1	
TNM stage			0.0869
<Ib	112	52	
≥Ib	70	18	
